# Characterization of Chicken-Derived Single Chain Antibody Fragments against Venom of *Naja Naja Atra*

**DOI:** 10.3390/toxins10100383

**Published:** 2018-09-21

**Authors:** Chi-Hsin Lee, Sy-Jye Leu, Yu-Ching Lee, Chia-I Liu, Liang-Tzung Lin, Pharaoh Fellow Mwale, Jen-Ron Chiang, Bor-Yu Tsai, Chi-Ching Chen, Ching-Sheng Hung, Yi-Yuan Yang

**Affiliations:** 1 School of Medical Laboratory Science and Biotechnology, College of Medical Science and Technology, Taipei Medical University, Taipei 11031, Taiwan; chihsine@msn.com (C.-H.L.); chliu@tmu.edu.tw (C.-I.L.); pmwale@medcol.mw (P.F.M.); 2 Graduate Institute of Medical Sciences, College of Medicine, Taipei Medical University, Taipei 11031, Taiwan; cmbsycl@tmu.edu.tw (S.-J.L.); ltlin@tmu.edu.tw (L.-T.L.); 3 Department of Microbiology and Immunology, School of Medicine, College of Medicine, Taipei Medical University, Taipei 11031, Taiwan; 4 The Center of Translational Medicine, Taipei Medical University, Taipei 11031, Taiwan; ycl@tmu.edu.tw; 5 Center for Research, Diagnostics and Vaccine Development, Centers for Disease Control, Ministry of Health and Welfare, Taipei 11561, Taiwan; jrc@cdc.gov.tw; 6 Navi Bio-Therapeutics Inc., Taipei 10351, Taiwan; boryutsai@navibio.com.tw; 7 Department of Pathology and Laboratory Medicine, Landseed Hospital, Taoyuan 32449, Taiwan; chen3971@landseed.com.tw; 8 Department of Laboratory Medicine, Wan Fang Hospital, Taipei Medical University, Taipei 11696, Taiwan; oryx@w.tmu.edu.tw; 9 Core Laboratory of Antibody Generation and Research, Taipei Medical University, Taipei 11031, Taiwan

**Keywords:** *Naja naja atra (NNA)*, venom proteins, IgY antibodies, phage display technology, single-chain variable fragment (scFv) antibody

## Abstract

Traditional, horse-derived antivenin is currently the most efficient treatment against snake bites. However, it is costly and has unpredictable side effects. Thus, alternative, cost-effective strategies for producing antivenin are needed. In this study, we immunized hens with inactivated NNA venom proteins from the cobra *Naja naja atra* (NNA). Purified yolk IgY antibodies showed specific anti-NNA binding activity comparable to that of the equine-derived antivenin. We used phage display technology to generate two antibody libraries containing 9.0 × 10^8^ and 8.4 × 10^8^ clones with a short or long linker, respectively. The phage ELISA indicated that anti-NNA clones displaying single-chain variable fragments (scFv) were significantly enriched after biopanning. The nucleotide sequences of the light and heavy chain genes of 30 monoclonal scFv antibodies were determined and classified into six groups with the short linker and nine groups with the long linker. These scFv clones specifically bound to NNA proteins but not to venom proteins from other snakes. Their binding affinities were further determined by competitive ELISA. Animal model studies showed that anti-NNA IgY antibodies exhibited complete protective effects, while a combination of scFv antibodies raised the survival rates and times of mice challenged with lethal doses of NNA venom proteins.

## 1. Introduction

Snake envenomation is considered a neglected tropical disease and is an often-discussed medical issue, especially in tropical regions [1,2]. The World Health Organization (WHO) indicated that 2.5 million venomous snake bites result in approximately 125,000 deaths every year. Since snake bites can occur in remote locations or be managed without medical attention, case numbers are most likely underestimated [1,3]. Snake venom contains a mixture of proteins with various functions that induce paralysis, death, and prey digestion [4]. Globally, cobras (*Naja* spp.) in the family *Elapidae*, are venomous snakes, and this includes the subspecies *Naja naja atra* (NNA), which is found in Taiwan. Venom proteins from *N. n. atra* (NNA proteins) contain a complex of components with different homologues, including phospholipase A2, snake venom metalloproteinases, and cysteine-rich secretory proteins, which exhibit various biological activities, such as neurotoxicity and cardiotoxicity (also called cytotoxicity) [5]. These components act synergistically or autonomously to induce various syndromes, such as local tissue swelling, tissue necrosis, and heart failure, that can only be treated by amputation or otherwise result in the death of victims [6]. Because of their strong toxicity, development of therapeutic agents against different venom components is limited. To date, equine antivenin has been the most frequently used therapy for treating venomous snake bites.

Immunotherapy, including antibody applications, has been an effective therapy for a broad range of conditions, such as cancers and snakebites [2,7]. To date, the most effective treatment against snakebites is the administration of antivenin, generated in hyperimmunized horses and containing numerous antibodies that recognize many different epitopes, which neutralize the toxic activity of the venom components. However, generation of equine antivenin in horses is costly and labor-intensive and associated with several potential side effects [8]. To attenuate the costs and the side effects related to mammal producers, hens might be an alternative source of immunoglobulin Y (IgY) antibodies [9]. There are several advantages to using hens as immunization hosts for antibody production, such as minimal husbandry expenses and the relatively small amount of antigens needed for immunization [10]. In addition, IgY has numerous virtues, such as cost-effective purification, non-invasive extraction procedures, no activation of the mammalian complement systems, and no interactions with Fc fragments that result in inflammatory responses [11]. Many studies have indicated that using IgY for passive immunization might be a good substitute therapeutic approach [12,13,14]. Moreover, chickens, rather than other animals, are increasingly used to produce specific monoclonal antibodies (MAbs) [15]. Since MAbs recognize one particular epitope, their specificity is high, rendering them suitable for the development of wound exudate diagnostic tests to determine the type of snake bites of victims [16,17]. MAbs with neutralizing activities against the toxicity of venom proteins could additionally become novel candidates for the development of antibody drugs for snake envenomation.

It was reported that phage display antibody technology can be used not only in MAbs generation but also in developing targeted therapies [18]. This novel technology is a fast and economical method for generating and selecting specific MAbs compared to hybridoma technology. The latter requires sophisticated processes and expensive materials [19]. In addition, single-chain variable fragments (scFv) can be readily cloned and expressed in prokaryotic cells using phage display technology. ScFv antibodies maintain both variable regions of heavy and light chains linked by a flexible peptide linker, and yet retain the specific binding activity of the parental IgG [20]. Although the neutralizing activity of monoclonal scFv antibodies might be less effective than polyclonal IgY antibodies when used against snake venom, a mixture of large quantities of monoclonal scFv antibodies may have the potential to remarkably reduce symptoms, augment survival rates, and prevent death [21]. In the majority of cases, snakebites are difficult to immediately identify because they present similar characteristics among species. Thus, MAbs, due to their highly specific nature, can precisely determine the type of snakebite and thus provide a better assessment of treatment choices.

In an attempt to evaluate an alternative strategy for neutralizing the toxicity of venom proteins, and to develop reagents for the rapid diagnosis of snakebites, we used attenuated NNA venom proteins administered to chickens to produce specific polyclonal IgY, while monoclonal scFv was obtained from the antibody libraries constructed by phage display technology. These IgY and scFv antibodies were assessed for their binding activities to six snake venom proteins and for their neutralization activities to determine their protective efficacy by injecting mice with a minimum lethal dose (MLD) of NNA venom proteins. These model IgY and scFv antibodies were created with the hope that they could potentially be applied to the future development of alternative rapid diagnostic and therapeutic reagents for snake envenomation.

## 2. Results

### 2.1. Characterization of Polyclonal Anti-NNA IgY Antibodies

**Figure 1:**
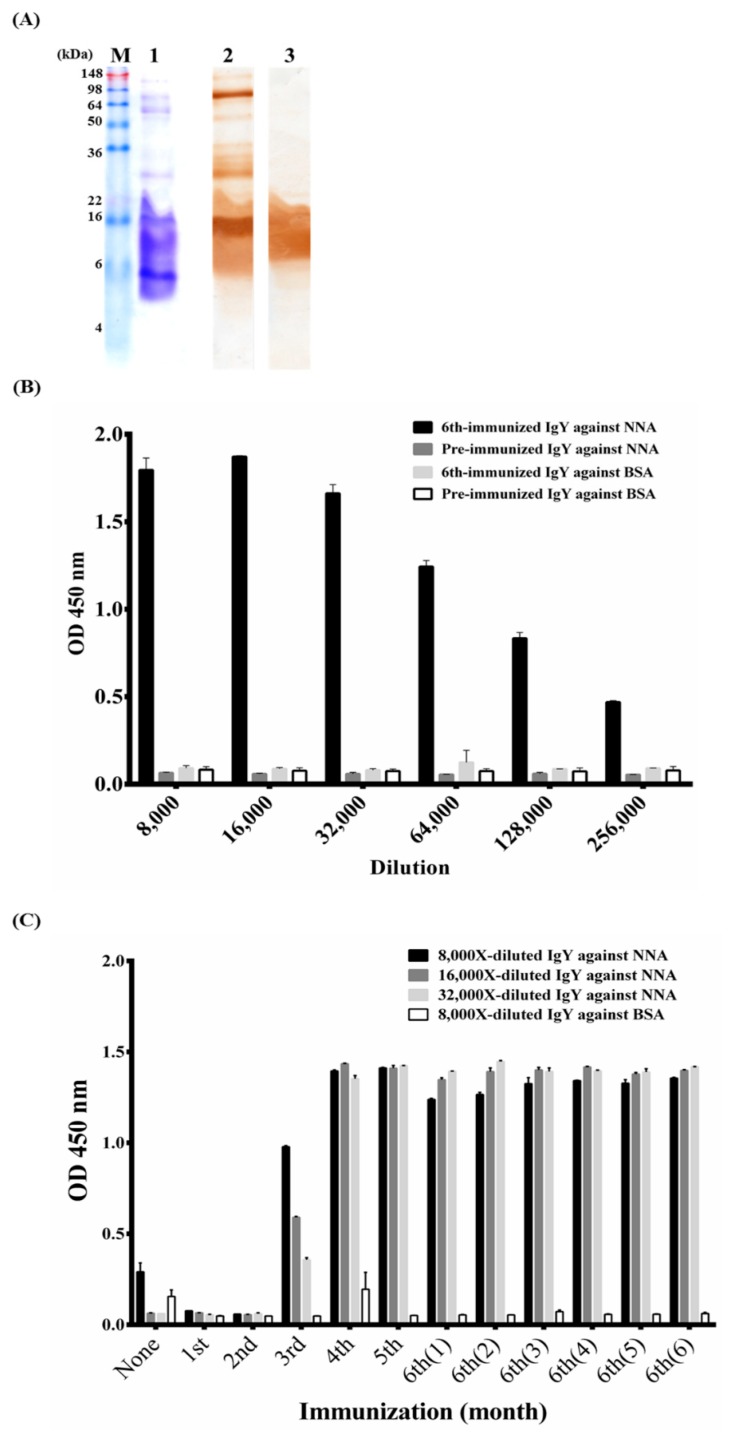
Characterization of partially purified immunoglobulin Y (IgY) antibodies from *Naja naja atra* (NNA)-immunized chickens. (**A**) NNA venom proteins separated by sodium dodecyl sulfate polyacrylamide gel electrophoresis (SDS-PAGE) were stained with Coomassie blue dye (lane 1). Later, equal amounts of proteins were blotted onto PVDF papers and detected using polyclonal IgY antibodies from the chickens immunized 6 times (lane 2) or equine-derived antivenin (lane 3). (**B**) Both preparations of polyclonal IgY antibodies were diluted (8000× to 256,000×) and checked for their specific binding activities to NNA proteins or BSA, respectively. (**C**) The anti-NNA IgY antibody response was monitored for 6 months after the 6th immunization (6th (6)). The results showed that specific anti-NNA IgY antibodies were elicited and remained at a significant level for a period of at least 6 months. Lane M was the protein standard.

NNA venom proteins were analyzed by sodium dodecyl sulfate polyacrylamide gel electrophoresis (SDS-PAGE) before chicken immunization. SDS-PAGE showed that NNA venom is composed of complex proteins, the majority of which have molecular weights less than 22 kDa. Additional, though less abundant, proteins with molecular weights of approximately 30, 60, 80 and 120 kDa were also observed, as shown in Figure 1A, lane 1. Polyclonal IgY from chickens immunized 6 times was purified to test its binding activity to NNA proteins. Western blots showed that anti-NNA IgY antibodies were generated effectively and recognized the lower molecular weight proteins, shown in Figure 1A, lane 3, with recognition patterns comparable to those of equine-derived antivenin, as seen in Figure 1A, lane 2. However, the horse-derived antivenin recognized several larger venom proteins, with molecular weights over 22 kDa, that were not recognized by the chicken-derived IgY antibodies. We further examined the binding specificity of the purified polyclonal IgY antibodies against NNA venom proteins. The results showed that the purified polyclonal IgY antibodies (64,000-fold diluted) bound strongly to NNA proteins (ODs > 1.0) without reacting with BSA, as seen in Figure 1B. In contrast, the preimmunization IgY antibodies showed minimal reactivity (ODs < 0.1) with NNA proteins and BSA, as seen in Figure 1B. We also monitored the progress of the chickens’ humoral response. IgY antibodies purified at different stages of immunization were analyzed by ELISA. The results showed that a significant humoral response was induced after the 4th immunization, which then reached a plateau stage that was steady for at least 6 months, as seen in Figure 1C. Taken together, the results indicate that polyclonal IgY antibodies against NNA venom proteins were effectively produced in chickens.

### 2.2. Antibody Library Construction

Total RNAs were extracted from the spleens of hyperimmunized chickens to synthesize cDNA copies. The copies were used as templates to amplify the V_L_ and V_H_ fragments of immunoglobulins using PCR. The amplified products were joined and extended by overlapping PCR to form single-chain variable fragments (scFv) approximately 750 bps long and containing a short or long peptide linker (data not shown). The recombinant PCR products were ligated into pComb3X vectors, cloned, and electroporated into *Escherichia coli* (*E. coli*) to construct two scFv antibody libraries. The resulting libraries were estimated to contain 9.0 × 10^8^ phages with short linkers (scFv-S) and 8.4 × 10^8^ phages with long linkers (scFv-L). After infecting *E. coli* with an M13 helper phage, the resulting phages were utilized for subsequent biopanning.

### 2.3. Selection of Monoclonal Anti-NNA scFv Antibodies

**Figure 2:**
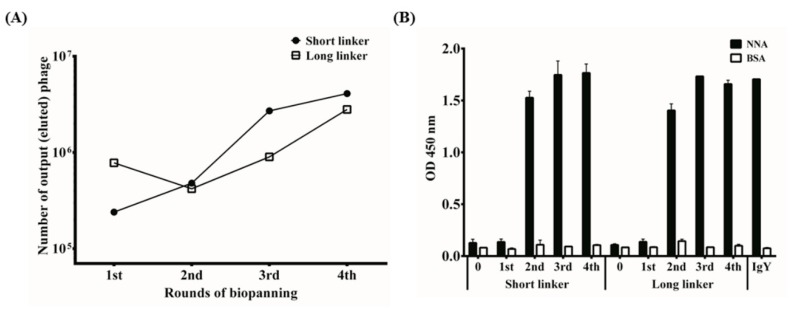
Biopanning and characterization of anti-*Naja naja atra* (NNA) single-chain variable fragments (scFv)-expressing phage libraries. (**A**) Two antibody libraries displaying scFv antibodies were established as described in the text. Recombinant phages were eluted to determine the number of colony-forming units (cfu) after each biopanning. (**B**) The recombinant phages (10^11^–10^12^ plaque forming units, pfu) after being amplified in *Escherichia coli* (*E. coli*) were analyzed by ELISA to test their binding activities to NNA proteins or BSA. Anti-NNA immunoglobulin Y (IgY) antibodies were partially purified and used as a positive control. In accordance with our previous studies, a significant increase in O.D. values after the 1st panning suggested that the phages expressing scFv antibodies against NNA venom proteins were enriched.

Recombinant phages displaying specific anti-NNA scFv antibodies were enriched through four rounds of biopanning (Figure 2A). There were approximately 4 × 10^5^ colony-forming units (cfu) of eluted phages in the scFv-S library after the 1st panning, which increased steadily to 5 × 10^6^ cfu after the 4th panning. By comparison, there were approximately 9 × 10^5^ cfu of eluted phages from the scFv-L library after the 1st panning, which decreased slightly after the 2nd panning, finally increasing to 3 × 10^6^ cfu after the 4th panning. The exact reasons for the titer difference between panning processes were not known. However, similar results were also observed in our previous studies (data not shown), suggesting that phages with anti-NNA activities were generously enriched by the biopanning procedures. The results were confirmed by phage ELISA. The anti-NNA binding activities of the amplified phage clones after each panning were determined by ELISA. IgY antibodies with anti-NNA activities were included as a control. As shown, the NNA-binding activity of recombinant phages greatly increased in the 2nd panning and plateaued thereafter. Little reactivity to BSA was measured, as shown in Figure 2B.

### 2.4. Sequencing Analysis of Anti-NNA scFv Antibodies

**Figure 3:**
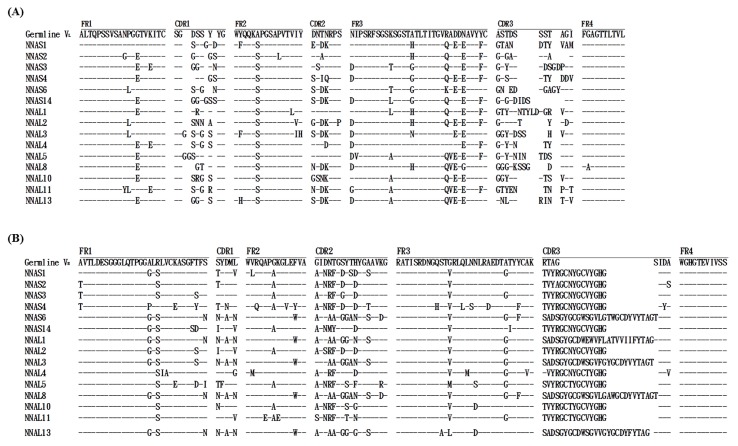
Sequence comparison of anti-*Naja naja atra* (NNA) single-chain variable fragments (scFv) V_L_ and V_H_ genes with chicken germline genes. Amino acid sequences were deduced from the nucleotide sequences of 15 representative anti-NNA scFv clones (6 NNAS with a short linker and 9 NNAL with a long linker) and aligned to those from the V_L_ (**A**) and V_H_ (**B**) of the chicken germline gene, respectively. Sequence gaps were represented by blank spaces to maximize the alignment. Identical amino acid residues were indicated by dashes (-). The boundaries of the framework regions (FRs) and complementarity-determining regions (CDRs) were denoted above the amino acid sequences of the germline genes.

Total DNA after the 4th panning was purified and transformed into TOP10F’ *E. coli*. Twenty clones were randomly chosen from the scFv-S and scFv-L libraries to express scFv antibody molecules. Thereafter, 15 clones expressing scFv antibodies from each library were selected to determine the nucleotide sequences of the V_L_ and V_H_ genes. Their amino acid sequences were deduced and aligned with those from the chicken immunoglobulin germline gene, shown in Figure 3. The results indicated that six distinct groups of scFv clones containing short linkers were identified and shown as NNAS1 (3/15; 20%), NNAS2 (5/15; 40%), NNAS3 (3/15; 20%), NNAS4 (1/15; 6.67%), NNAS6 (1/15; 6.67%) and NNAS14 (1/15; 6.67%). Similarly, 9 groups of scFv clones containing long linkers were identified and shown as NNAL1 (1/15; 6.67%), NNAL2 (1/15; 6.67%), NNAL3 (2/15; 13.33%), NNAL4 (4/15; 26.67%), NNAL5 (2/15; 13.33%), NNAL8 (2/15; 13.33%), NNAL10 (1/15; 6.67%), NNAL11 (1/15; 6.67%) and NNAL13 (1/15; 6.67%), as seen in Table 1. Moreover, the alignment also showed higher mutation rates in the complementarity-determining regions (CDRs) than in the framework regions (FRs). Noticeably, CDR3s of the V_L_ and V_H_ genes contained 32–60% and 50–75% of the mutation rates, respectively, shown in Table 2. The results indicated that anti-NNA scFv clones were elicited and selected by an antigen-driven antibody response in immunized chickens.

As shown in the V_L_ genes of the anti-NNA clones, CDR1s had 7–10 amino acids, CDR2s had 7 amino acids, and CDR3s had 9–16 amino acids as well as the highest mutation rates (40–100%), shown in Figure 3A and Table 2. In the V_H_ genes of the anti-NNA clones, CDR1s and CDR2s had 5 and 17 amino acids, respectively, identical to those of the germline gene. By contrast, all CDR3s had either 18 or 30 amino acids, while those from the germline gene had only 8. Noticeably, the heavy chain sequences of the CDR3s in NNAS1, NNAS3, NNAS14 and NNAL2 were identified as TVYRGCNYGCVYGHGIDA, and the sequences in NNAL10 and NNAL11 contained an N to T change. The heavy chain sequences of the CDR3s in NNAS2, NNAS4, NNAL4 and NNAL5 also showed a high degree of homology to TVYRGCNYGCVYGHGIDA, as seen in Figure 3B. Given these observations, we strongly suggest that the highly conserved CDR3 sequences may be essential to the binding of anti-NNA scFv antibodies with NNA venom proteins.

### 2.5. Expression, Purification and Characterization of Selected scFv Antibodies

**Figure 4:**
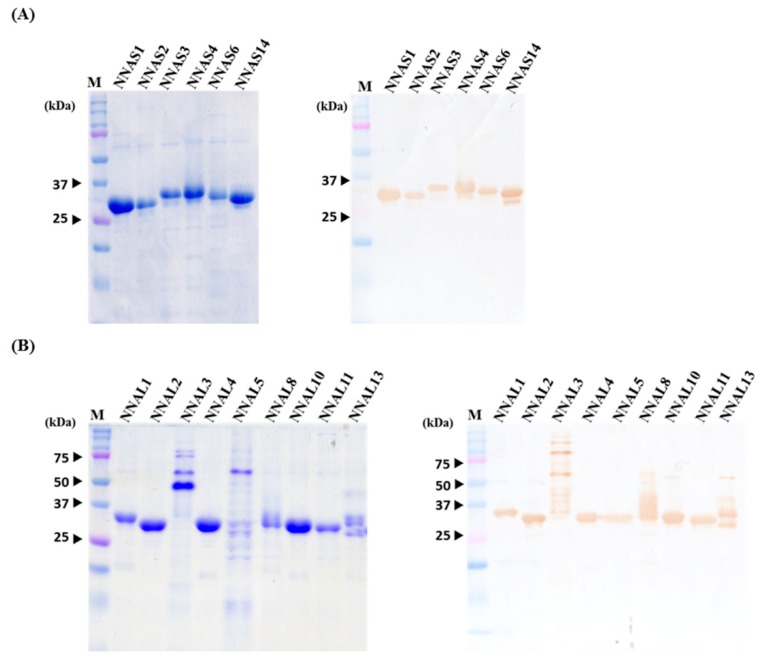
Purification and characterization of 15 anti-*Naja naja atra* (NNA) single-chain variable fragments (scFv) antibodies. (**A**) The selected scFv clones were induced to express by the addition of IPTG, purified using Ni^2+^ Sepharose and detected on sodium dodecyl sulfate polyacrylamide gel electrophoresis (SDS-PAGE) (**left panels** in **A** and **B**). (**B**) After transfer onto PVDF membranes, these scFv antibodies were further identified using goat anti-chicken light chain antibodies, followed by HRP-conjugated anti-goat IgG antibodies (**right panels** in **A** and **B**). Lanes NNAS1~NNAS14 and NNAL1~NNAL13 represented the anti-NNA scFv antibodies containing short and long linkers, respectively.

After overnight IPTG induction, the recombinant scFv antibodies were purified and analyzed by SDS-PAGE and Western blots, as described above. The major bands with slight differences in molecular weights (25–37 kDa) were visualized, suggesting that the scFv antibodies were successfully expressed and purified, as seen in Figure 4A,B, left panels,. The purified scFv was confirmed using anti-chicken light chain antibodies, shown in the right panels of Figure 4A,B. However, the scFv antibodies from NNAL3 and NNAL5 clones showed protein bands with larger molecular weights, 50–75 kDa, on SDS-PAGE and Western blots. We do not know the identity of these proteins but consider them to be the aggregated form of the scFv antibodies. The problem could occasionally be solved by adding 6 M urea to the solution during the antibody purification process.

### 2.6. Specific Binding of Anti-NNA scFv Clones

**Figure 5:**
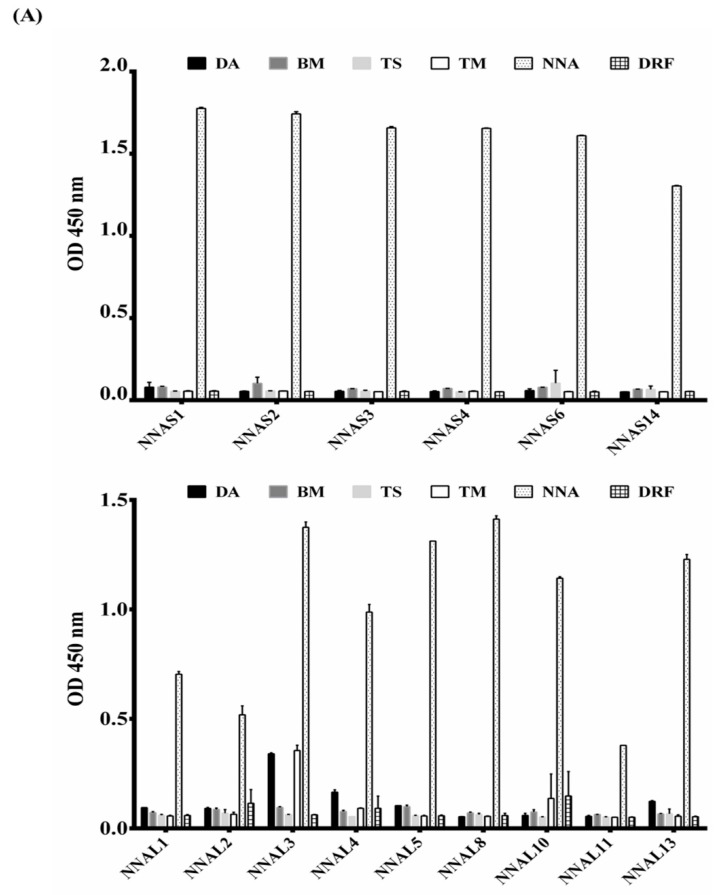

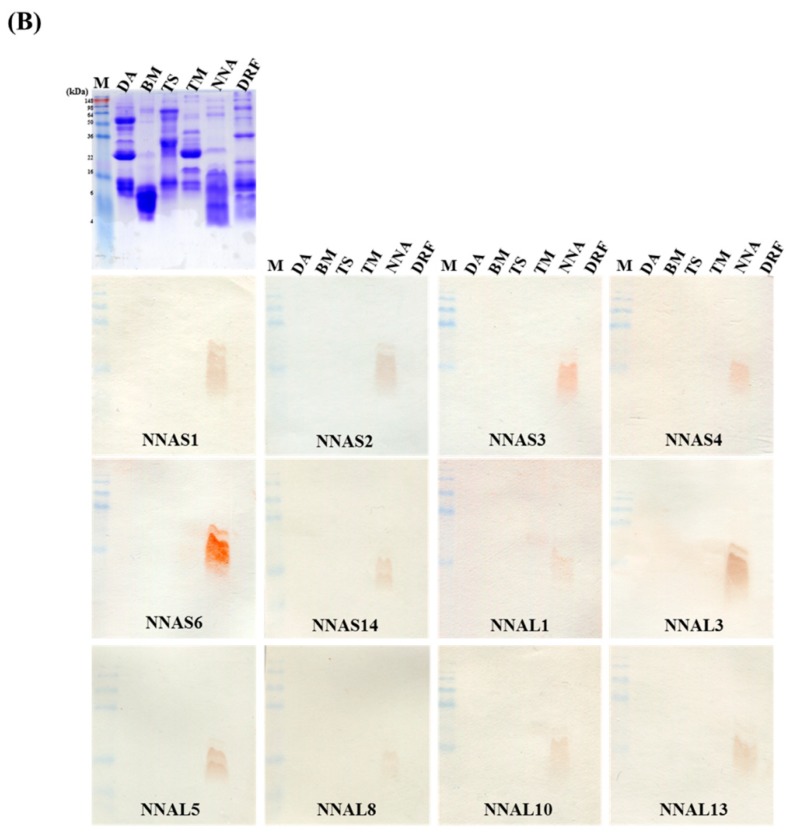
Specificity of anti-*Naja naja atra* (NNA) single-chain variable fragments (scFv) clones against six snake venom proteins. (**A**) The purified scFv antibodies were analyzed for their binding activities to venom proteins from *Bungarus multicinctus* (BM), *Deinagkistrodon acutus* (DA), *Trimeresurus stejnegeri* (TS), *Trimeresurus mucrosquamatus* (TM), *Daboia russelii formosensis* (DRF) and NNA fixed on ELISA wells. (**B**) The potential proteins recognized by these scFv antibodies were further identified using Western blots. No binding signal was detected by NNAL2, NNAL4 and NNAL11 scFv antibodies and thus not shown here.

All 15 purified scFv antibodies, immobilized on ELISA wells or PVDF papers, were used to detect venom proteins from six dominant snake species in Taiwan (DA, BM, TS, TM, NNA and DRF). ELISA results indicated that the purified scFvs displayed different degrees of binding activities to NNA venom proteins, as seen in Figure 5A. NNAL1, NNAL2 and NNAL11 scFvs showed lower binding activities (ODs < 1.0), while the remaining scFvs showed higher binding activities (ODs > 1.0). As anticipated, the majority of these scFvs showed no reactivity against venom proteins from DA, BM, TS, TM and DRF (ODs < 0.2), except for NNAL3, which showed a slight cross-reactivity with the DA and TM venom proteins (ODs = 0.2–0.4). Furthermore, the binding specificities of these scFvs were testified on Western blots. NNAS1, NNAS2, NNAS3, NNAS4, NNAS6, NNAS14, NNAL1, NNAL3, NNAL5, NNAL8, NNAL10 and NNAL13 recognized a group of low molecular weight NNA proteins (<16 kDa) to varying extents, as seen in Figure 5B, while NNAL2, NNAL4 and NNAL11 showed no detectable signals (data not shown). In addition, no reactivity to venom proteins from DA, BM, TS, TM and DRF was detected. Intriguingly, it was observed that NNAS6 showed moderate binding activity on ELISA but the most significant recognition signals on Western blots. Taken together, significant correlations in the binding activities of these scFv antibodies, seen in both immunological assays, indicated highly specific NNA venom protein recognition and possible recognition of the conserved antigenic epitopes present in a group of venom proteins.

### 2.7. Inhibition Assay by Competitive ELISA

**Figure 6:**
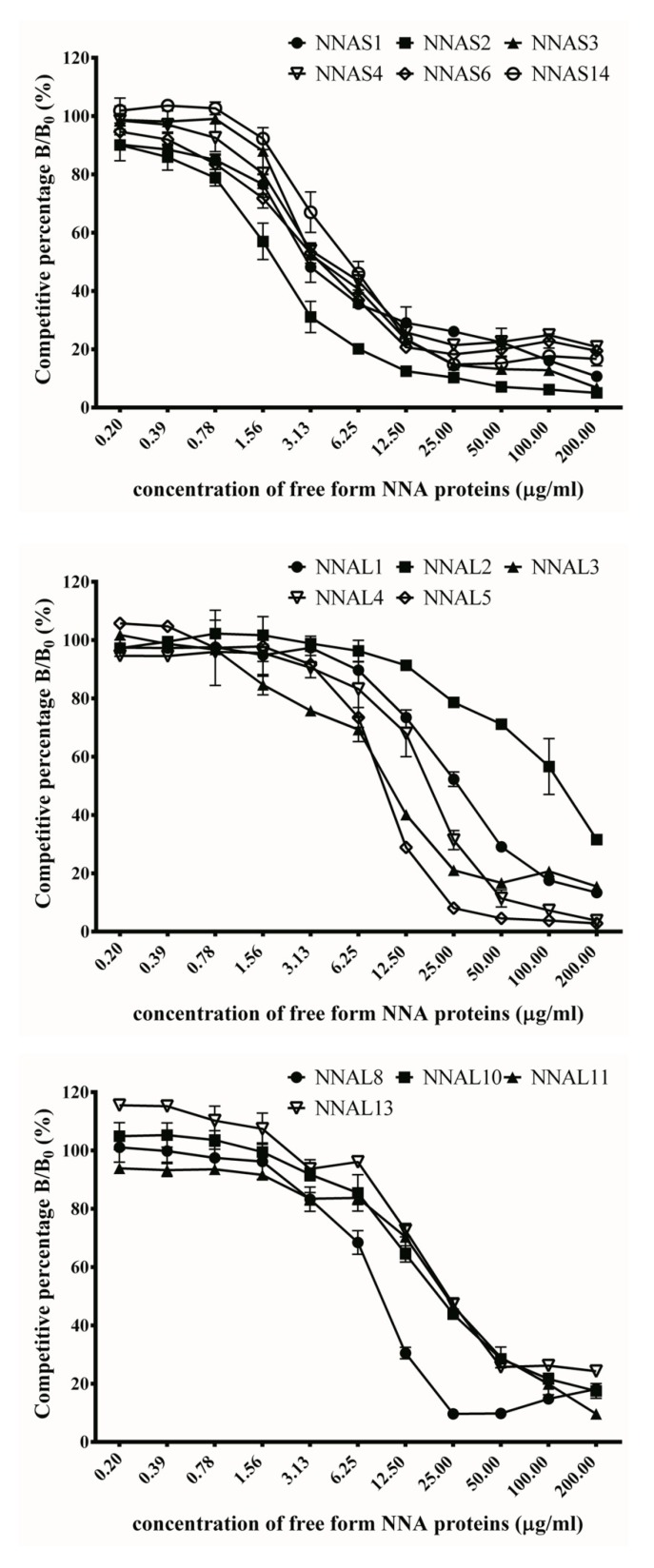
Competitive inhibition of anti-*Naja naja atra* (NNA) single-chain variable fragments (scFv) clones against NNA venom proteins. Purified scFv antibodies were preincubated with serially diluted venom proteins (200–0.2 μg/mL) and then loaded onto the ELISA plates coated with venom proteins (see the text for detail). The bound scFv antibodies in the presence or absence of soluble venom proteins were represented as B/B_0_ to show the inhibitory percentage of the binding specificity. The results are presented in duplicate.

The competitive ELISA method was performed to further confirm the binding activities of anti-NNA scFv antibodies. Each scFv antibody was separately incubated with the free form of NNA venom proteins and then loaded to the ELISA plate immobilized with NNA proteins. The percentage of inhibitory effect was calculated using optical readings of the absence over the presence of different concentrations of the free form of NNA proteins. The dissociation constant (*Kd*) of each scFv was calculated using the Klotz plot method. As shown, the binding activities of these scFvs were significantly suppressed in a dose-dependent manner, as seen in Figure 6. It is noteworthy that the *Kd* values of NNAS scFv antibodies were 1.3 to 4.7 × 10^−7^ M, while the values NNAL scFv antibody clones were 7.4 × 10^−7^ to 2.3 × 10^−6^ M, as shown in Table 3, respectively. These combined results demonstrate that NNAS2 (1.3 × 10^−7^ M) had the strongest affinity while NNAL1 (2.3 × 10^−6^ M) was the most impotent binder.

### 2.8. Neutralization Assay of Anti-NNA IgY and scFv Antibodies

**Figure 7:**
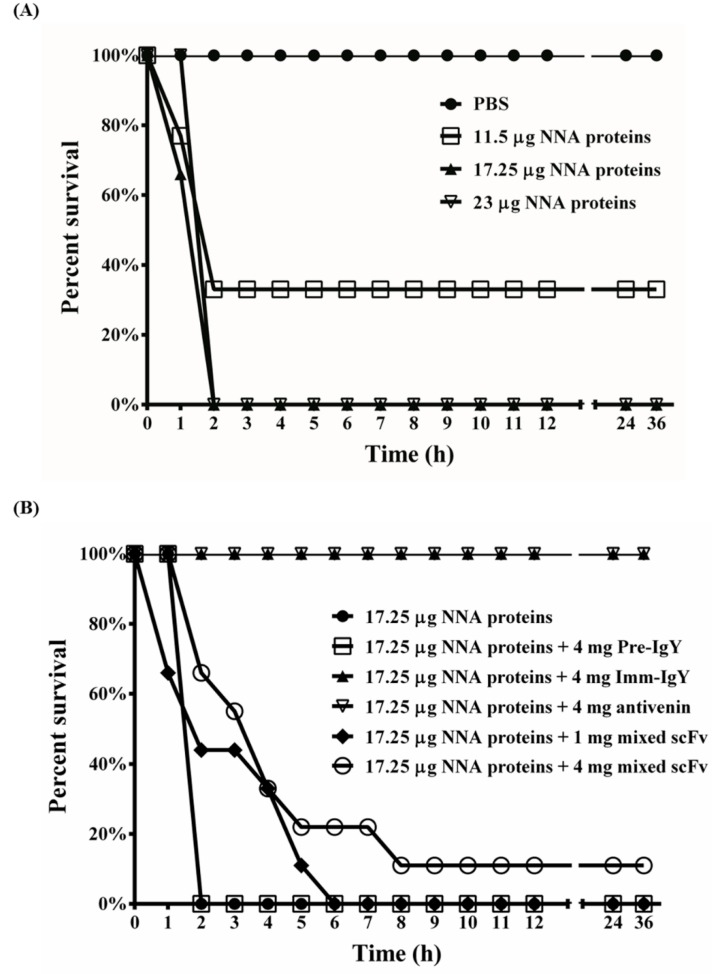
Protective effects of anti-*Naja naja atra* (NNA) antibodies in an animal model. ICR mice were randomly assigned to groups of 9 and intraperitoneally injected with various doses of NNA venom proteins (11.5, 17.25 and 23 μg) to determine the minimal lethal dosage (MLD) (**A**) Mice injected with PBS were used as controls. Purified IgY antibodies from preimmunized hens (Pre-IgY), hens immunized 6 times (Imm-IgY), equine-derived antivenin (Antivenin), or a mixture of 15 anti-NNA scFvs (1 or 4 mg) were separately incubated with 17.25 μg of NNA venom proteins at 37 °C for 1 h. Each mixture was later intraperitoneally injected into mice (**B**) The tested mice were monitored, and the observed results were recorded hourly for 36 h.

First, we injected mice intraperitoneally with 11.5, 17.25 or 23 μg of NNA venom proteins to determine the MLD, as seen in Figure 7A. The data indicated that administration of 11.75 μg of NNA proteins caused the death of 2 mice within 1 h and 4 within 2 h; 3 mice survived with no obvious abnormalities. In contrast, all mice treated with either 17.25 or 23 μg of NNA proteins expired within 2 h, while 100% of PBS-treated mice survived, as shown in Figure 7A. Thus, 17.25 μg of NNA proteins was taken to be the 1× MLD and was used in subsequent experiments. The results in Figure 7B shows that injection with 4 mg of IgY from preimmunized hens (Pre-IgY) mixed with 1× MLD of NNA proteins caused 100% mortality of the mice within 2 h. In comparison, all mice survived when injected with a mixture of 1× MLD of NNA proteins and 4 mg of anti-NNA IgY from 6th-immunized hens (Imm-IgY) or equine-derived antivenin. It was noted that survival time was significantly prolonged in mice treated with a mixture of 1× MLD of NNA proteins and 1 mg of 15 anti-NNA scFv antibodies. Two mice (22%) were completely protected throughout the study when treated with a mixture of 1× MLD of NNA proteins and 4 mg of 15 anti-NNA scFv antibodies (Figure 7B). The combined data suggest that a mixture of 15 NNA scFv antibodies could provide partial neutralizing activity and greatly increase the survival time for mice injected with NNA venom proteins.

## 3. Discussion

Snake envenomation is a common public health problem that affects a large number of lives globally, especially in tropical regions. Standard procedures for the generation of equine antivenin are expensive and the complexity of such antivenin can cause serious side effects to the patients [8]. Moreover, the collection and preparation of snake venom proteins to immunize larger animals such as horses are difficult. The WHO and US Food and Drug Administration (FDA) has recommended a reduction in animal experiments and efforts to decrease the pain of animal test subjects [22]. Therefore, we intended to develop alternative therapeutic and rapid diagnostic reagents against snake envenomation that are more cost-effective, which would be important for underdeveloped countries and remote areas. Hens are readily available in most regions in the world, cheap to obtain and easily maintained in confined spaces. It is common to harvest more than 40 g IgY/hen/year, which contains up to 10% antigen-specific antibodies and persists at higher titers for longer periods [23,24,25]. Unlike human IgG, IgY antibodies are not recognized by rheumatoid factors and therefore do not form immune complexes or activate mammalian complement systems [26,27]. Most importantly, only a small quantity of antigens is needed to induce a significant antibody response in hens, which is particularly suitable for generating antibodies against a limited source of immunogens, such as snake venom proteins [28]. In this study, 500 μg of glutaraldehyde-inactivated NNA venom proteins were used as immunogens to obtain 10–15 mg of specific anti-NNA IgY antibodies per egg, 1–2 months after immunization. By contrast, approximately 20 mg of immunogen is required to elicit a significant antibody response in horses, according to WHO guidelines [29,30]. Intriguingly, the low molecular weight proteins in NNA venom, which are normally considered to trigger relatively limited immunogenicity, elicited a stronger IgY antibody response than the large proteins did, as shown in Figure 1. Moreover, the produced anti-NNA IgY exhibited complete inhibitory activity, as seen in Figure 6B. Several studies have made similar observations [31,32,33].

The phage display system is a widely applied technology for producing MAbs against a variety of antigens, including anti-venom antibody proteins [21,34,35,36]. Previous reports indicated that it is more feasible to acquire better quality and quantity antibody libraries using fewer cDNA templates transcribed from the spleen RNA of immunized hens rather than nonimmunized (naïve) ones. In accordance with this observation, we constructed two libraries containing 9.0 × 10^8^ and 8.4 × 10^8^ transformants from hyperimmunized chickens. It should be noted that it required only 2 rounds of biopanning to obtain the anti-NNA antibody clones, as indicated in Figure 2B, whereas it generally takes more than 4–6 rounds to obtain specific antibodies when using libraries from naïve chickens. Furthermore, it is commonly agreed that the hybridoma system takes at least 3 months, on average, to generate MAbs, while the phage display system needs only 2–3 weeks after the last immunization of the chickens [37]. More importantly, producing monoclonal antibodies or fragments with transfected cells is safer than via hybridoma technology because of the lower genetic stability of hybridoma cell lines.

Sequence analysis indicated that the 30 anti-NNA scFv antibodies selected after the last biopanning were classified into six and nine groups of antibodies with short (NNAS) or long linkers (NNAL), respectively, as shown in Figure 3 and Table 1. The identical V_H_ and V_L_ genes were predominantly used by 40% of the NNAS or 26.67% of NNAL scFv antibodies, as represented by NNAS2 and NNAL4, respectively. Of note, 2 highly conserved motifs consisting of TVYRGCNYGCVYGHGIDA or SADSGYGCGWSGVLGTWGCDYVYTAGTIDA sequences were identified in the CDR3 domains of the V_H_ genes. CDR3 has been considered the most variable and important region with anti-antigen properties, especially in the V_H_ gene [38]. Thus, the conserved structure may have an important role in antigen recognition or the accurate folding of these anti-NNA scFv antibodies. This observation is in accordance with a previous report, which suggested that the V_H_ fragments of IgY are crucial for their binding specificity [39]. In addition, the amino acid sequences deduced in the study contained a great degree of variation compared to those of the chicken germline. In particular, all six CDR domains had higher mutation rates than did FR domains, in which CDR3 domains of the V_H_ and V_L_ genes had the most variation, as demonstrated in our previous studies [31,32,40]. In addition to amino acid sequence variation, the length of the CDR3 region of the V_H_ gene may also have a significant impact on the binding activity of the antibodies. It is well documented that the CDR3 regions of the V_H_ gene normally contain 8–32 amino acid residues (mean 16.2 ± 3.2) and are similar to those of humans (5–37 amino acids, mean 16.1 ± 4.1) but longer than those of mice (5–26 amino acids, mean 11.8 ± 2.4) [39]. The same study reported that in chickens 89% of CDR3s were 15–23 amino acids in length, and our clones, being 18 amino acids long, were in this range, except for NNAS6, NNAL1, NNAL3, NNAL8, and NNLA13, which were longer, as shown in Figure 3B. Otherwise, these scFvs had similar V_H_ CDR3s including NNAS1, NNAS3, NNAS4 (except for one amino acid difference), NNAS14 and NNAL2, which had almost the same CDR3 in the V_H_, and NNAL10 and NNAL11, which had the same CDR3 in the V_H_. We considered that these similar CDR3 sequences in the V_H_ are important factors acting against NNA proteins. However, NNAL2 had weaker binding activity than NNAS1, NNAS3, NNAS4 and NNAS14, and NNAL11, which also showed lower binding activities than NNAL10 on ELISA and on Western blot assays. We presume that a difference in the V_L_s or the lengths of the linkers affected the conformation of these scFvs, which resulted in the reduction of the binding activities to NNA proteins.

All anti-NNA scFv antibodies showed different binding activities specific to venom proteins from the cobra NNA, but not to those from other snakes such as DA, BM, TS, TM and DRF, seen in Figure 5A, among which NNAL1, NNAL2, NNAL4 and NNAL11 showed weaker binding activities (OD < 1.0). It is well documented that only strong antigen-binders would be selected after several rounds of rigorous biopanning, but the reason(s) that binders with lower binding activities were obtained was not known. The results may be ascribed to the difference between the conformation of scFv antibodies expressed on the surface of M13 viral particles and the conformation of the soluble form within *E. coli* host cells. Western blot analysis showed that these purified scFv antibodies recognized NNA proteins with molecular weights smaller than 22 kDa, which make up more than 90% of the NNA crude venom and are considered to be phospholipase A2 (14–17 kDa), neurotoxins (7–10 kDa), and cardiotoxins (6.5–7.0 kDa), seen in Figure 5B, as demonstrated in previous reports [5,41,42]. Generally, it is believed that the phospholipase A2, neurotoxins and cardiotoxins in the NNA venom collected from around the world are the three major components of lethal venom [43,44]. In Thailand, neurotoxins with various molecular weights are the major lethal components of Thai cobra (*Naja kaouthia*) venom, and could be partially neutralized using scFv antibodies [21,42]; however, cardiotoxins or phospholipase A2 from the Indian cobra may also exhibit a lethal effect after envenomation [45,46]. Several studies suggested that different isoforms of cardiotoxins or neurotoxins may be the lethal components in certain species of cobra snakes in Taiwan [43,47]. Taken together, the results from these studies indicate that the components of venom proteins and their concentrations are distinct to the species of cobra, leading to the aforementioned difference in the venomous enzymatic activities of each venom. In this context, the phenomenon is also true for the venom proteins with various levels of lethal toxicity collected from cobra NNA in different regions in Taiwan [5]. Thus, we proposed that low molecular weight venom proteins may act synergistically to cause a number of syndromes such as tissue swelling, heart and breathing failures, systemic problems, and even death when no treatment is administered. This idea was supported by the results of the neutralization assay shown in Figure 7. Mixtures of purified anti-NNA scFvs provided partial protective effects in mice administered with a lethal dose of NNA venom proteins. In vivo studies showed that these scFvs recognized only a group of neutralization epitopes from neurotoxins, phospholipase A2 or cardiotoxins, leading to partial inhibition of lethal envenomation. In addition, the less protective effects of scFv antibodies may be ascribed to their shorter half-life (0.1–0.5 days) compared to those of IgY (4.1 days) and antivenin (2 days). However, additional studies, including mass spectrometric analysis, are essential to further confirm the identity of the targeted venom protein and the underlying inhibitory mechanisms of these anti-NNA scFv antibodies.

While we used the rodent mortality model to demonstrate the protective effect of the developed scFv, there are limitations to this analysis, notably, the differential impact of toxins in humans and rodents. Some toxin groups, such as alpha neurotoxins, have been documented to be less important in inducing paralysis in humans than in rodents [48], while rodents are observed to be resistant to snake procoagulant toxins which, conversely, are clinically important to humans [49]. For this reason, our analysis of NNA-induced mortality in mice may not accurately reflect the same conditions in humans, and hence provides a limited functional examination of the scFvs in vivo. Additional, more clinically focused functional assays, including chick biventer assays for neurotoxicity and myotoxicity coupled with in vitro anti-coagulant or pro-coagulant assays using human plasma, and viability assays using human cell lines, may provide a clearer functional examination of the scFv’s protective activity against NNA venom. Given that anti-NNA polyclonal IgY and monoclonal scFv antibodies provided full and partial protection against envenomation in mice, respectively, we believe that additional anti-NNA scFv antibodies recognizing more neutralization epitopes on the venomous constituents could be identified from the antibody libraries constructed in this study shown in Figure 7. Thus, all the anti-NNA IgY and scFvs combined experimentally would have the potential to be developed into rapid diagnostic and therapeutic reagents against lethal snakebites. To avoid the human anti-chicken antibody (HACA) response, these anti-NNA scFv antibodies require further optimization and modification by the replacement of Fc portion (chimeric antibodies) or grafting of complementarity-determining regions (CDR) (humanized antibodies) to improve their binding activities and reduce their immunogenicity as reported by Tsurushita et al. [50].

## 4. Materials and Methods

### 4.1. Animals

The experimental protocols used for the chickens and mice were approved by the Institutional Animal Care and Use Committee of Taipei Medical University. Female Leghorn (*Gallus domesticus*) hens aged 6 months and ICR mice weighing 12–14 g were kept in the animal core facility at TMU. (Ethical approval code: LAC-2017-0253; valid on 15 November 2017 to 14 November 2019).

### 4.2. Chicken Immunization and IgY Purification

We dissolved the NNA protein, generously offered by the Centers for Disease Control (CDC) in Taiwan, in phosphate-buffered saline (PBS). Protein identities were confirmed by sodium dodecyl sulfate polyacrylamide gel electrophoresis (SDS-PAGE). We mixed 100 μg of NNA venom protein with 0.125% glutaraldehyde at room temperature for 1 h. The mixture was emulsified with 1/2 volume of Freund’s complete adjuvant and injected intramuscularly into hen legs for the first immunization. Then, we mixed 80 μg of NNA proteins in incomplete adjuvant for the subsequent immunizations, administered at intervals of 7 days. We collected the eggs daily until the sixth immunization. The purifications of polyclonal IgY antibodies were carried out using dextran sulfate, as reported in a previous study [51].

### 4.3. Library Construction

Antibody libraries were constructed as described previously [19]. We sacrificed the hens after the final immunization. We extracted total RNA from spleen tissue homogenized in 5 mL of Trizol buffer for cDNA synthesis (Invitrogen, Carlsbad, CA, USA), based on the manufacturer’s instructions. The synthesized cDNA products were used for amplifying the variable regions of light chains (V_L_) and heavy chains (V_H_) of immunoglobulin genes using chicken-specific primers. The primers CSCVHo-F/CSCG-B, CSCVHo-FL/CSCG-B and CSCVK/CKJo-B (60 pmole/each) were used for the amplification of V_H_ with a short linker (V_H-S_), V_H_ with a long linker (V_H-L_) and V_L_ genes, respectively. The primers CSC-F/CSC-B (60 pmole/each) were used to amplify the full-length scFv gene. The PCR was programmed according to the following conditions: 94 °C for 5 min, 30 cycles of 94 °C for 15 s, 56 °C for 15 s, 72 °C for 90 s, and a final incubation step at 72 °C for 10 min. The PCR products were pooled, concentrated by ethanol-precipitation and purified by gel extraction. After *SfiI* (New England Biolabs, Ipswich, MA, USA) digestion at 50 °C for 5 h, scFv genes were ligated into pComb3X vectors at 23 °C overnight. The recombinant products were then purified and transformed into ER2738 *Escherichia coli* (*E. coli*) by electroporation at 3.0 kV (MicroPulser, Bio-Rad, Hercules, CA, USA). We inoculated a small aliquot of the transformed bacteria to LB agar plates to determine the library size. We added the remaining cultures to 100 mL of super broth (SB) containing 10 μg/mL tetracycline (Tet)/50 μg/mL of ampicillin, and then added 10^12^ plaque forming units (pfu) of VCS-M13 helper phages, then incubated the culture at 37 °C overnight. We collected the amplified recombinant phages in the supernatant after centrifugation at 3000× *g* for 10 min at 4 °C and precipitated on ice for 30 min with 4% polyethylene glycol 8000 (*w*/*v*) and 3% NaCl (*w*/*v*). The pellet was centrifuged at 15,000× *g* for 15 min at 4 °C and resuspended in PBS containing 1% BSA and 20% glycerol. We determined the phage titers and stored at −20 °C until use.

### 4.4. Biopanning

We carried out the biopanning in ELISA wells for four rounds. Briefly, we coated the NNA venom proteins in 1× PBS at 0.5 µg/well, incubated overnight at 4 °C and then blocked with 3% BSA in 1× PBS at 37 °C for 1 h. We added 10^11^–10^12^ pfu of recombinant phages to the wells, and incubated at 37 °C for 2 h. We removed the unbound phages with PBST (1× PBS containing 0.05% Tween 20) and eluted the bound phages with 0.1 M glycine-HCl (pH 2.2), then neutralized with 2 M Tris-base buffer. Thereafter, we infected ER2738 bacteria with the eluted phages for amplification. We determined the phage titers using a plaque forming assay. We cultured the infected *E. coli* bacteria overnight and collected the amplified phages for the next round of biopanning. Four to six rounds were typically necessary to enrich the specific phages displaying anti-NNA scFvs.

### 4.5. Expression and Purification of scFv Antibodies

We purified total phagemid DNA from the 4th round of biopanning and transformed TOP10F’ *E. coli* to express anti-NNA scFvs. The overnight culture of each randomly chosen clone (1 mL) was diluted 100× in super broth (SB) medium containing 20 mM MgCl_2_ and 50 μg/mL of Amp, which was further incubated at 37 °C for 8 h (or until it reached an OD reading of approximately 0.6). We empirically added various concentrations of isopropyl-β-D-thiogalactopyranoside (IPTG) (0.1–1.0 mM) overnight induction of scFv antibody expression. Then, we collected the bacterial cultures by centrifugation at 3000 revolution per minute (rpm), resuspended them in 1/10 volume of histidine (His) binding buffer (20 mM sodium phosphate, 0.5 M NaCl, 20 mM imidazole, pH 7.4), and lysed the cells by sonication. After one additional centrifugation (as above), we purified the His-tagged scFvs in the supernatant in a column containing Ni^2+^ Sepharose, based on the manufacturer’s instructions (GE Healthcare BioSciences AB, Uppsala, Sweden). We washed the column 3 times with His-binding buffer and eluted the scFvs by adding His-elution buffer. Finally, we used dialysis in PBS to remove the salts from the eluted solution and concentrated the affinity-purified scFvs with Ultra4 Centrifugal Filter Units (Merck Millipore, Darmstadt, Germany).

### 4.6. Sequencing

We determined the nucleotide sequences of the V_L_ and V_H_ genes of 15 randomly chosen scFv-expressing clones from each antibody library using an ompseq primer (5′-AAGACAGCTATCGCGATTGCAGTG-3′) and an ABI 3730 XL autosequencer (Applied Biosystems, Foster City, CA, USA). We deduced the amino acid sequences of the scFvs, which were aligned with those of the chicken immunoglobulin germline genes, to delineate the structures of the frameworks (FWs) and the complementarity determining regions (CDRs) using the BioEdit alignment program [19].

### 4.7. Western Blotting

We separated NNA venom proteins by SDS-PAGE, which were transferred to polyvinylidene fluoride (PVDF) papers and incubated with PBS containing 5% skim milk at 25 °C for 1 h. We incubated the membranes with 1:1000 diluted equine-derived anti-NNA antivenin (a gift from CDC, Taiwan) at 25 °C for 1 h. We removed the unbound antibodies with PBST and added horseradish peroxidase (HRP)-labeled goat anti-horse Fab antibodies (Jackson ImmunoResearch, West Grove, PA, USA). After 3 wash steps, we detected the bound horse anti-NNA antibodies by the addition of diaminobenzidine (DAB). Similarly, we examined the binding activity of polyclonal IgY antibodies from the eggs of pre- and post-immunized hens using HRP-labeled donkey anti-chicken IgY (Jackson ImmunoResearch, West Grove, PA, USA). To further test their binding specificities, we incubated the purified anti-NNA scFvs (5 μg/mL) with membranes fixed with venom proteins from *Bungarus multicinctus* (BM), *Deinagkistrodon acutus* (DA), *Trimeresurus stejnegeri* (TS), *Trimeresurus mucrosquamatus* (TM), *Daboia russelii formosensis* (DRF), and NNA. Then, we detected the bound anti-NNA scFvs using goat anti-chicken light chain IgG (Bethyl Laboratories, Montgomery, TX, USA), followed by HRP-labeled donkey anti-goat IgG (Jackson ImmunoResearch, West Grove, PA, USA). We performed the steps for blocking, washing and signal detection as reported above.

### 4.8. Enzyme-Linked Immunosorbent Assay (ELISA) and Competitive ELISA

We coated the ELISA wells with NNA venom proteins and BSA (10 µg/mL) at 4 °C overnight and blocked them with 1× PBS containing 5% skim milk at 37 °C for 1 h. Then, we incubated 2× serially diluted (500× to 256,000×) polyclonal IgY from the preimmunized or immunized hens at 37 °C for 1 h. After 3 washes, we added the HRP-labeled donkey anti-chicken IgY and incubated for 1 h at 37 °C. After washing (as above), we measured the binding activities by adding 3,3′,5,5′-tetramethylbenzidine (TMB). We stopped the reactions with 1 N HCl and recorded the optical density readings at 450 nm. For the phage ELISA, we incubated 10^11^–10^12^ pfu of phage particles from each panning with NNA venom proteins coated on the wells and detected binding with HRP-labeled mouse anti-M13 antibodies (GE Healthcare Bio-Sciences, Marlborough, MA, USA). To test their binding activities, we added the purified anti-NNA scFv antibodies to the ELISA wells coated with venom proteins from BM, DA, TS, TM, NNA, and DRF. Finally, we used the goat anti-chicken IgY light chain and HRP-labeled donkey anti-goat IgG antibodies for visualization.

For the competitive ELISA, we mixed NNA venom proteins in a 2-fold dilution series (200 to 0.2 μg/mL) with an equal volume of individual scFvs (10 μg/mL) at 25 °C for 1 h. Then, we added the mixtures to the wells with NNA venom proteins and incubated at 37 °C for 1 h. We performed the blocking, washing and signal detection steps as described above. The amounts of NNA proteins required to reach 50% inhibitory effects on the binding activities of the scFv antibodies were measured based on the competitive ELISA results. Then, the equation B_0_/(B_0_ − B) = 1 + *Kd*/A_0_ was used to calculate the *Kd* values, as reported by Friguet et al. [52]. B_0_, B and A_0_ represent the O.D. reading in the absence of the free form of the NNA proteins, the O.D. reading in the presence of the free form of the NNA proteins, and the concentration of the NNA proteins, respectively. All the data were shown as the mean ± SD from independent duplicated experiments.

### 4.9. Neutralization Activity of scFv Antibodies

We performed the neutralization assay according to the recommendations of the WHO protocol. We randomly allocated ICR mice into groups of nine. We intraperitoneally injected mice with 11.5, 17.25 or 23 μg of NNA venom proteins in 200 µL of 1× PBS to determine the minimum lethal dose (MLD). We included PBS with no NNA proteins as a negative control. Thereafter, we injected 11.5 µg of venom proteins (1 MLD) in 200 µL of 1× PBS mixed with IgY from preimmunized hens, IgY from hens immunized 6 times (4 mg/each, ~111 µM), a mixture of fifteen anti-NNA scFvs (4 mg, ~740 µM) and equine-derived antivenin (4 mg, ~182 µM) to evaluate their inhibitory effects on mice. We recorded the conditions of the mice every hour for 36 h after injection.

## Figures and Tables

**Table 1:** Classification of anti-*Naja naja atra* (NNA) single-chain variable fragments (scFv) clones.

	Short Linker			Long Linker		
	V_L_	V_H_	Percentage	V_L_	V_H_	Percentage
**Group 1**	1, 7, 15	1, 7, 15	20%	1	1	6.67%
**Group 2**	2, 5, 8, 9, 10, 13	2, 5, 8, 9, 10, 13	40%	2	2	6.67%
**Group 3**	3, 11, 12	3, 11, 12	20%	3, 15	3, 15	13.33%
**Group 4**	4	4	6.67%	4, 6, 9, 14	4, 6, 9, 14	26.67%
**Group 5**	6	6	6.67%	5, 7	5, 7	13.33%
**Group 6**	14	14	6.67%	8, 12	8, 12	13.33%
**Group 7**				10	10	6.67%
**Group 8**				11	11	6.67%
**Group 9**				13	13	6.67%

V_L_: Variable region of the light chain; V_H_: Variable region of the heavy chain.

**Table 2:** Amino acid mutation rates of the light and heavy chain genes of the anti-NNA scFv antibodies.

Region	CDR1	CDR2	CDR3	Total CDRs	FR1	FR2	FR3	FR4	Total FRs
**V_L_**	13~50%	0~57%	40~100%	32~60%	0~15%	6~29%	9~25%	0~10%	8~14%
**V_H_**	0~60%	24~53%	83~90%	50~75%	3~13%	7~29%	6~19%	0%	5~16%

CDRs: Complementarity determining regions; FRs: Framework regions; V_L_: Variable region of the light chain; V_H_: Variable region of the heavy chain.

**Table 3:** Calculated dissociation constant (*Kd*) values of anti-*Naja naja atra* (NNA) single-chain variable fragments (scFv) antibodies.

Clone	Inhibition of 50% Binding of Total Protein (μg/mL)	*Kd* Values (M)
**NNAS1**	4.07 ± 0.127	3.1 ± 0.009 × 10^−7^
**NNAS2**	1.73 ± 0.255	1.3 ± 0.191 × 10^−7^
**NNAS3**	4.77 ± 0.373	3.6 ± 0.283 × 10^−7^
**NNAS4**	4.93 ± 0.067	3.7 ± 0.050 × 10^−7^
**NNAS6**	4.19 ± 0.329	3.1 ± 0.248 × 10^−7^
**NNAS14**	6.29 ± 1.233	4.7 ± 0.919 × 10^−7^
**NNAL1**	30.58 ± 0.840	23 ± 0.645 × 10^−7^
**NNAL2**	128.59 ± 9.549	*
**NNAL3**	11.89 ± 0.929	8.9 ± 0.700 × 10^−7^
**NNAL4**	18.48 ± 4.040	*
**NNAL5**	10.50 ± 0.643	7.9 ± 0.481 × 10^−7^
**NNAL8**	9.87 ± 0.508	7.4 ± 0.382 × 10^−7^
**NNAL10**	21.42 ± 1.012	16 ± 0.778 × 10^−7^
**NNAL11**	23.18 ± 5.571	*
**NNAL13**	27.97 ± 0.56	21 ± 0.424 × 10^−7^

* not calculated due to the lack of signal detection on Western blots.
